# Expression of the Autoimmune Regulator Gene and Its Relevance to the Mechanisms of Central and Peripheral Tolerance

**DOI:** 10.1155/2012/207403

**Published:** 2012-10-22

**Authors:** Roberto Perniola

**Affiliations:** Neonatal Intensive Care, Department of Pediatrics, V. Fazzi Regional Hospital, Piazza F. Muratore, 73100 Lecce, Italy

## Abstract

The autoimmune polyendocrine syndrome type 1 (APS-1) is a monogenic disease due to pathogenic variants occurring in the autoimmune regulator (*AIRE*) gene. Its related protein, AIRE, activates the transcription of genes encoding for tissue-specific antigens (TsAgs) in a subset of medullary thymic epithelial cells: the presentation of TsAgs to the maturating thymocytes induces the apoptosis of the autoreactive clones and constitutes the main form of central tolerance. Dysregulation of thymic *AIRE* expression in genetically transmitted and acquired diseases other than APS-1 may contribute to further forms of autoimmunity. As *AIRE* and its murine homolog are also expressed in the secondary lymphoid organs, the extent and relevance of AIRE participation in the mechanisms of peripheral tolerance need to be thoroughly defined.

## 1. Introduction

Pathogenic variants in the autoimmune regulator (*AIRE*) gene cause the autoimmune polyendocrine syndrome type 1 (APS-1), also called autoimmune polyendocrinopathy-candidiasis-ectodermal dystrophy (APECED), an autosomal recessive disease characterized by immunological disturbances such as difficulty to eradicate surface candidiasis and autoimmunity to various organs, mainly endocrine glands [1, 2]. *AIRE* is located in the region q22.3 of chromosome 21 [3], and its cloning dates back to the second half of the nineties [4, 5]. APS-1 is a rare model of monogenic autoimmune disease and in this quality provides unequivocal insights into the pathogenesis of more complex diseases of analogous nature [6, 7].

Due to its biochemical properties, AIRE protein activates the transcription of genes encoding for tissue-specific antigens (TsAgs) in a subset of medullary thymic epithelial cells (mTECs): this phenomenon, called promiscuous gene expression (PGE), is finalized to the promotion of central (thymic) tolerance [8]. The last step of the process is represented by the deletion (negative selection) of T-cell clones bearing T-cell receptors (TCRs) with critical degree of specificity for the corresponding TsAgs [9].

Nonetheless, the nature and extent of AIRE action remain unclearly defined. A rigorous mapping of *AIRE* gene expression is fundamental to the dissection of the protein role, but the current data show several incongruities, presumably due to differences in tissue substrate and sensitivity of the methods utilized.

Here an extensive review of the studies pertaining to the argument is reported, with an additional look at the relation between disturbances in *AIRE* expression and diseases other than APS-1 in human field and animal models.

## 2. The Initial Studies

### 2.1. Initial Mapping of AIRE Expression. 

The first two studies, which dealt with *AIRE* cloning and searched for *AIRE* mRNA in bulk tissue samples by Northern blotting (NB), gave disagreeing responses [4, 5]. Although the thymus showed the highest level of positivity in both cases, one research group found a further weak positivity only in lymph-nodal, fetal liver, and appendix samples [4], while the other one signaled also a moderate to strong positivity in the samples from bone marrow, spleen, peripheral blood lymphocytes (PBLs), and organs such as thyroid, pancreas, adrenal gland, and testis, in other words the endocrine glands targeted by autoimmunity in APS-1 [5].

### 2.2. Identification of AIRE-Expressing Cell Lineages

Later, the same research groups stated that the highest amount of *AIRE* mRNA and AIRE protein could be identified, by in situ hybridization (isH) and immunohistochemistry (IHC), in rare cells scattered in the medulla and subcapsular area of the thymus, or buried in the Hassall's corpuscles [10, 11].

In immunofluorescence (IF), these cells were seen to express surface markers such as cytokeratins, molecules of the class-II major histocompatibility complex (MHC-II), and the clusters of differentiation CD80, CD86, and CD40, and for this reason were recognized as mTECs, the main components of the thymic stroma. A minority of thymic AIRE^+^ cells expressed CD11c and CD83, which identify mature dendritic cells (DCs) of myeloid lineage [10].

In the secondary lymphoid organs, one research group detected AIRE only in the medulla and paracortical area of the lymph nodes, in the spleen and fetal liver: similarly to thymic DCs, lymph-nodal AIRE^+^ cells expressed CD83, suggesting a common identity [10].

In contrast, the other group observed significant staining of medullary thymocytes, lymph-nodal and splenic red-pulp lymphocytes, and PBLs; other leukocyte populations, such as neutrophilic granulocytes and monocytes, were also AIRE^+^. The results, obtained by IHC and immunocytochemistry (ICC), were confirmed by quantitative real-time reverse transcriptase-polymerase chain reaction (RT-PCR) [11].

By IF, freshly isolated PBLs were positively stained for AIRE in the study of Rinderle et al. too [12].

## 3. *AIRE* Expression in the Cells of the Immune Response and in Nonlymphoid Organs

The initial studies set in motion the debate, still outstanding, on the existence of cell lineages, complementary to mTECs, in which *AIRE* would be expressed, and the related meaning. As expected, such debate primarily deals with *AIRE* expression in the cells of the immune response.

### 3.1. AIRE Expression in the Monocyte/DC Lineage

Measurable amounts of *AIRE* mRNA and AIRE protein were evidenced in CD14^+^ cells sorted from peripheral blood, and in monocyte-derived DCs through in vitro differentiation [13–15], with isolated exceptions [16]. One of these research groups found *AIRE* mRNA also in plasmacytoid DCs isolated directly from the peripheral blood [15]. Members of the mitogen-activated protein kinase (MAPK) family would be involved in the signal-transduction pathway allowing *AIRE* expression in the monocyte/DC lineage [14, 17]. Interestingly, it was observed that DC maturation is paralleled by increasing AIRE levels and ordinary up-regulation of several genes [14].

Later, Poliani et al. detected *AIRE* expression in frozen samples of lymph nodes and gut-associated lymphoid tissue (GALT) from adult subjects, while the fetal samples were negative: the cells responsible for such positivity expressed surface markers typical of mature DCs [18].

### 3.2. AIRE Expression in the Lymphocyte Lineage

*AIRE* expression in the lymphocyte lineage remains quite uncertain: reappraising and partly correcting their previous findings [13], Nagafuchi et al. found, by RT-PCR, *AIRE* mRNA in PBLs belonging to the CD4^+^ T-cell subset; the transcription level increased under antigen- or cytokine-mediated activation [19]. Another Japanese research group detected *AIRE* mRNA in thymic B lymphocytes and double-positive (DP) CD4^+^CD8^+^ thymocytes, while in the peripheral blood it was restricted to B lymphocytes only [20].

### 3.3. AIRE Expression in Nonlymphoid Organs

In two of the above studies, a large set of human organs was assayed by RT-PCR and IHC, and the authors agreed that organs of the endocrine, cardiovascular, respiratory, gastrointestinal, genitourinary, and nervous systems are either consistently negative or negligibly positive for *AIRE* expression [16, 18]. Based on the observation that mTECs and cancer cells share PGE, Klamp et al. included RT-PCR of samples from human cancers, but no *AIRE* expression was found [16].

So, to recapitulate, only Finnish researchers detected *AIRE* mRNA in human tissues such as endocrine glands and other nonlymphoid organs, albeit the cell lineages detaining such property were not defined [5].

### 3.4. Unexpected Localizations

An unexpected localization of *AIRE* mRNA was found by Harris et al., who studied two unrelated APS-1 adolescents with chronic abnormalities of endochondral ossification, characterized by irregular and radioopaque metaphyses, subjacent to the growth plates of long bones; *AIRE* expression, although not searched for in the bone samples of the patients, was assayed in the thymus, liver, and growth plates of healthy fetuses aborted at 13–18 weeks of gestational age, in chondrocytes and in two chondrosarcoma lines: in all examined tissues, *AIRE* expression resulted consistently active. In particular, in the growth plate of the knee, *AIRE* mRNA appeared at 15 weeks of gestational age and was still present at 18 weeks [21].

Similar considerations may be done for *AIRE* expression in epidermal keratinocytes, in keratinocytes of the outer and inner epithelial sheaths of the hair follicle, and in matrix melanocytes [22, 23]. At this level, AIRE is identifiable at 16 weeks of gestational age and colocalize with cytokeratin 17, a protein constitutive of the intermediate filaments [23].

The meaning of *AIRE *expression in chondrocytes and keratinocytes remains quite obscure. Intriguingly, Clark et al. had already proven that human skin cells (keratinocytes and fibroblasts), once cultured in a three-dimensional arrangement resembling the thymic architecture, have *AIRE* expression, synthesize a large set of TsAgs, and are able to perform a thymus-like function in de novo maturation and negative selection of T lymphocytes [24]. Currently, the demonstration that this observation may have some in vivo equivalence is lacking.

Findings pertaining to *AIRE* expression in human tissues are resumed in Table 1.

## 4. *Aire* Expression in the Mouse

Researches on the murine homolog (*Aire*, printed in lower case to avoid confusion) recapitulate most findings and incongruities encountered in dealing with human tissues, as resumed in Table 2.

### 4.1. Searching for Aire mRNA

Not surprisingly, the low level of *Aire* expression made some methods, such as NB, unsuitable to detect *Aire* mRNA even in the thymus [29, 30, 33].

On the other hand, RT-PCR gave consistently positive results on the thymus [25–31, 34], lymph nodes [25–27], spleen [25–29, 31], and liver [25, 26, 28].

Performing RT-PCR on the cells obtained by enzymatic digestion of whole thymus and spleen, and sorted by flow cytometry, Heino et al. found *Aire* mRNA in mTECs and, to a lesser degree, in DCs: further analysis of the latter revealed *Aire* transcription in two thymic (CD8*α* ^+^ and CD8*α* ^−^) and three splenic (CD4^+^CD8*α* ^−^, CD4^−^CD8*α* ^−^ and CD4^−^CD8*α* ^+^) subsets of myeloid and lymphoid lineage [26].

RT-PCR demonstrated higher sensitivity than isH that, when employed to map *Aire* expression on tissue sections, detected *Aire* mRNA only in rare foci of mTECs of murine embryos (from 14.5 days after conception), and young and adult mice [28, 29, 33].

On the other hand, a Finnish research group observed, by isH, an additional staining of a small number of medullary thymocytes, and of the lymph-nodal paracortical zone, the splenic red pulp, and immature bone marrow elements belonging to various cell lineages [25]. In the same study, RT-PCR detected *AIRE* mRNA in a remarkable number of organ samples [25].

### 4.2. Searching for Aire Protein

Aire protein was found by Western blotting (WB) in the only thymus [26, 28]; by IHC, Heino et al. observed that Aire^+^ cells belonged to a subset of mTECs, distributed among resting (60%) and activated (30%) elements, as revealed by CD95 and CD29, respectively. In embryonic thymus, Aire^+^ cells appeared at 14 days after conception [26]. In the same study, IHC was unable to stain Aire^+^ cells in any other tissue examined, albeit RT-PCR had detected *Aire* mRNA in the lymph nodes and spleen after the first round of amplification, and in the liver and various other organs after two rounds of the procedure [26].

Conversely, reproducing the results of RT-PCR, Halonen et al. found Aire^+^ cells in several organs [25]. In a following study, the authors strengthened these results by comparing tissue reactivity in wild-type and *Aire*-deficient (*Aire* ^−/−^) mice [32]. The findings were later supported by a UK research group [28].

It should be underlined that almost all cited studies utilized polyclonal antibodies (Abs) from mouse or rabbit to stain AIRE^+^/Aire^+^ cells in human and murine tissues, respectively, while the use of monoclonal Abs was rare and gave a restricted positivity [10, 18].

In this sense, Hubert et al., using rabbit monoclonal anti-Aire Abs, found Aire^+^ cells among murine mTECs only, albeit in the same study *Aire* mRNA had been detected also in thymic and splenic DCs [35]. Then the authors formulated an unifying theory, hypothesizing that the amount of *Aire* mRNA detected could be below the critical level useful to induce the translation into its protein, and that only monoclonal anti-Aire Abs would allow fine and unequivocal mapping of true *Aire* expression. It was not excluded that some extrathymic cells may express *Aire* in particular, not yet fully known, conditions [35].

### 4.3. Other Relevant Localizations

Two research groups identified, in the stroma of lymph nodes (and, in one instance, spleen), Aire^+^ cells that exhibited a CD45^−^CD80^−^CD86^−^MHC-II^+^ phenotype with further, albeit not coincident, epithelial-cell markers. Such cells were able to induce the deletion of CD8^+^ T-cell clones bearing TCRs specific for antigens encoded by Aire-dependent genes: the clones had been transferred into irradiated mice reconstituted with *β*_2_-microglobulin-deficient (*β*_2_-*m* ^−/−^) bone marrow to ensure that only radioresistant stromal cells of the secondary lymphoid organs could interact with them [36, 37].

Searching for *Aire* expression in lymph-nodal and splenic stroma by other research groups did not produce unequivocal results [31, 38–40]. The localization of Aire in secondary lymphoid organs may be connected to not yet defined mechanisms of peripheral tolerance integrating the thymic function by enlarging the set of controlled genes, deleting autoreactive T-cell clones that escape thymic deletion, or both [41, 42].

In another study, RT-PCR and IHC were used to demonstrate *Aire* transcription and translation in spermatogonia and early spermatocytes, where Aire would play a role in the program of early, scheduled apoptosis indispensable to the maintenance of germline stability [43].

Findings and controversies over the extrathymic expression of human *AIRE* and its murine homolog have been reviewed by Eldershaw et al. [44].

## 5. Regulation of *AIRE* Expression

### 5.1. Signal-Transduction Pathways

Besides to the integrity of the gene in itself, thymic *AIRE* expression requires that of two signal-transduction pathways enabling heterodimeric nuclear transcription factors known as NFs-*κ*B: NF-*κ*B1 includes a protein, p50 (from p105 precursor), and the transcription factor reticuloendotheliosis viral oncogene homolog A (RelA), while NF-*κ*B2 includes protein p52 (from p100 precursor) and another member of Rel family, RelB. In epithelial-cell lineages, NFs-*κ*B control cell proliferation, differentiation, and survival [45].

Thymic intercellular signaling promotes the pathways: at least three members of tumor necrosis factor (TNF)-receptor family represented on mTEC surface, namely CD40, receptor activator of NFs-*κ*B (RANK) and LT-*β* receptor (where LT stays for lymphotoxin), are able, by interaction with their respective partners on CD4^+^ thymocytes, namely CD40 ligand, RANK ligand and LT-*α*_1_ *β*_2_, and by means of TNF-receptor-associated factors (TRAFs), to initiate the cascade of reactions ending in NF-*κ*B activation [46–50].

### 5.2. Timing of AIRE Expression

*AIRE* expression is confined to a final stage of cell maturation, as shown in vitro and in vivo by the postmitotic status of murine Aire^+^ mTECs [51, 52]; in addition, Aire^+^ mTECs show a very limited life span [53, 54]. It is also indicative that Aire^+^ mTECs, because of their degree of differentiation, are highly sensitive to the drug-mediated ablation of the thymic medulla, and that their regeneration follows an invariant pattern [55].

It has been suggested that *AIRE* expression, and consequently that of AIRE-dependent genes, are strategically delayed just to allow a full T-cell responsiveness [56].

### 5.3. Modulation of AIRE Expression

Currently, we do not know whether the level of *AIRE* expression is genetically set, and whether metabolic, or environmental, or other agents are able to modulate it. This phenomenon, if determining the amount of TsAgs encoded by AIRE-dependent genes, could influence the chances of the autoreactive T-cell clones to encounter their targets and impact the efficiency of negative selection.

Studies on *Aire* ^−/−^ mice showed that the thymic expression of the genes dependent on Aire is quantitatively related to the amount of it, and that, in heterozygous (*Aire* ^+/−^) mice, intermediate level of mRNAs condition the number of autoreactive T-cell clones escaping thymic deletion [57]. This led the researchers to suggest that, in human field, the condition of heterozygosity for pathogenic *AIRE* variants could confer a risk for the onset of sporadic autoimmune diseases, when acting in synergy with other susceptibility factors. Actually, no data support this hypothesis.

Chen et al. found that, in the murine thymus, baseline *Aire* expression is related to the genetic background, as mTECs of nonobese diabetic (NOD) mice displayed lower levels of mRNAs from *Aire* and three Aire-dependent TsAg-encoding genes, when compared to mTECs of Balb/c mice [58]; Heino et al. had already found that Aire^+^ mTECs of NOD mice show an abnormal morphology [26].

Later, Venanzi et al. demonstrated that, in non-autoimmune-prone C57BL/6 mice, Aire activates more strongly the transcription of TsAg-encoding genes, and that the same genes are more severely downregulated in *Aire* ^−/−^ animals of the same strain. Unexpectedly, the percentage of Aire^+^ mTECs was higher in the thymus of NOD mice [59].

According to these findings, autoimmune-prone mice would show a less strict regulation of dependence on Aire, more than a deficient amount of it.

## 6. Thymic Diseases and *AIRE* Expression in Human Field

### 6.1. Severe Combined Immunodeficiency (SCID)

Omenn syndrome is characterized by peripheral expansion of oligoclonal T lymphocytes with autoreactive propensity. Impairment in various steps of T-cell maturation may cause the disease: the most frequent defect is caused by pathogenic variants in the recombinase-activating genes 1 and 2 (*RAG-1* and *RAG-2*, resp.) [60]. In all jawed vertebrates, RAG proteins induce a DNA rearrangement, called V(D)J recombination, that reassembles the exons encoding for the antigen-binding domains of TCRs from the native variable, diversity, and joining gene segments [61–63].

Some patients with Omenn syndrome and RAG deficiency have a marked decrease of circulating T and B lymphocytes, a condition referred to as T^−^B^−^SCID [64]. In either thymus or peripheral blood mononuclear cells (PBMCs) of patients suffering from these diseases, a substantial AIRE reduction was found [65–67]. In this sense, such forms of immunodeficiency, the classical SCID included, confirm the crucial role of AIRE in the mechanisms of central tolerance [68, 69].

A reasonable interpretation of what happens in these conditions leads to suppose that the thymi of the patients bearing genetically transmitted defects of the molecules involved in the developmental steps of T lymphocytes (with privileged reference to the construction of TCR diversity), show abnormalities of TEC differentiation, and consequently of *AIRE* expression, that are proportional to the timing of intervention of the same factors; hypomorphic variants of the related genes would result in more subtle disturbances [70–72].

### 6.2. Thymomas

Thymomas are rare tumors derived from TECs that are often associated with autoimmune diseases, mainly myasthenia gravis, caused by Abs to the acetylcholine receptor (AChR). It has been hypothesized that thymoma-associated AIRE deficiency may impair the tolerance to AChR and other antigens [73]. In facts although *AIRE* expression in thymomas is clearly decreased in terms of *AIRE* mRNA and AIRE^+^ cells, this datum does not correlate with the prevalence of myasthenia gravis [74, 75]. Thymoma patients do not exhibit the typical picture of APS-1 [76], albeit with isolated exceptions [77], but some resemblances between thymoma-associated myasthenia gravis and APS-1 exist [78, 79].

An interesting point of contact between APS-1 and thymomas is the presence of circulating Abs to various cytokines, such as interferons (IFNs) and interleukins (ILs): in the original article of Meager et al., sera from APS-1 patients showed high-titer neutralizing Abs to type-1 IFNs such as IFN-*α*, all subtypes included, and IFN-*ω*; IFN-*β*, another member of type-1 IFNs, as well as IFN-*λ*_1_, a subtype of type-3 IFNs, were less frequently targeted [80]. The same Abs were found in a large number of thymoma patients with myasthenia gravis, albeit the titer was significantly lower [80].

These findings led the authors to hypothesize that in abnormal thymic microenvironments made vulnerable by AIRE deficiency, the process of autoreactivity focuses early on molecules, such as type-1 IFNs, that result to be abundantly in loco available [81].

In a second time, Abs to the cytokines produced by Th17 subset of T-helper lymphocytes, namely IL-17A, IL-17F, and IL-22, were found in a high number of APS-1 patients; remarkably, the occurrence of such Abs in thymoma patients regarded mostly the restricted number of them that suffered from chronic mucocutaneous candidiasis, strengthening the similarities between the two diseases [82, 83].

In a further study, the same research group, utilizing a radioligand-binding assay, confirmed that Abs to IFN-*ω* occur in the totality of APS-1 patients and beat the prevalence of Abs to two subtypes of IFN-*α*, namely IFN-*α*_2_ and IFN-*α*_8_, that in turn are found in a high percentage of patients with thymoma-associated myasthenia gravis [84].

Leaving aside the autoimmune phenomena, the hypothesis that AIRE deficiency may contribute in thymomas to the tumor-promoting antiapoptotic features of TECs should not be discharged [85].

### 6.3. Down Syndrome

Down syndrome is characterized by thymic atrophy, and a decrease in AIRE^+^ cells was found in the thymus of subjects with Down syndrome who had undergone surgical thymectomy because of congenital heart malformations [86]. These data seem to deny that susceptibility to autoimmunity in Down syndrome would be a consequence of the precocious ageing.

## 7. Thymic Diseases and *Aire* Expression in Animal Models

### 7.1. Experimental Blocks in Thymocyte Maturation

Engineering animal models in which thymic organogenesis is disturbed provide a relevant contribution to the comprehension of the phenomena observed in the corresponding human diseases. A strategic choice is the block, at various stages of the process, of the thymocyte maturation, with the study of the related consequences on the architecture of the thymus in its entirety, and on the developmental steps of TECs: Tg*ε*26 and *Rag*-deficient (*Rag* ^−/−^) mice are examples of animal models utilized in such studies.

Tg*ε*26 mouse expresses a high number of the invariant CD3-*ε* chain belonging to TCR complex, and its thymocytes are blocked at DN1-DN2 stages, where DN stays for double-negative and indicates a CD4^−^CD8^−^ condition, with subdivision based on the progressive expression of CD44 and CD25. *Rag* ^−/−^ mouse recalls the most frequent defect causing Omenn syndrome: as reported, RAG/Rag proteins are indispensable to create TCR diversity and an adequate T-cell repertoire; the consequence of their deficiency is an impaired thymocyte maturation, with a block at DN3 stage [87].

A prototypical study in this field was that of Zuklys et al.: as seen above, *Aire* mRNA and Aire protein are recovered in murine embryos at 14–14.5 days after conception, slightly anticipating DN3 stage of thymocyte maturation. Consistently with these data, the authors found that thymi from Tg*ε*26 mice lacked an orthodox three-dimensional TEC network, and *Aire* mRNA could not be detected; conversely, the block of thymopoiesis in *Rag*-2^−/−^ mice altered only partially the thymic compartmentalization, and the related mTEC differentiation and *Aire* expression [88].

As well as in human field, following studies suggest that the degree of thymic abnormalities, *Aire* expression included, depends on how precociously the factors damaged by pathogenic variants of the encoding genes act in the construction of TCR diversity [89–92].

### 7.2. Experimental Defects in NF-*κ*B Signal-Transduction Pathways

Several studies have taken in account various murine constitutional and experimentally induced defects involving the molecules that participate to the signal-transduction pathways enabling NFs-*κ*B, to elucidate the impact of each step impairment on mTEC properties, with particular regard to *Aire* expression [26, 88, 93–117]. A detailed report of such articles goes beyond the scope of the present work, but, as indicated, excellent reviews are available [46–50].

### 7.3. Other Experimental Diseases Targeting the Thymus

Also protozoan infections, such as that from *Trypanosoma cruzi*, target the thymus and are able to cause its atrophy: Morrot et al. studied a murine model of Chagas disease and found that thymic expression of *Aire* and TsAg-encoding genes was preserved, albeit this condition was accompanied by early release of activated T lymphocytes into the periphery [118].

## 8. Conclusions and Future Remarks

It is definitively proven that the highest level of *AIRE* expression, as well as that of its murine homolog, is seen in a subset of mTECs. The low-level transcription in thymic DCs is presumably finalized to increase the availability of TsAgs to be presented to the autoreactive T-cell clones.

The detection of *AIRE* mRNA in nonlymphoid organs remains questionable and could be due to the presence, in bulk tissue samples, of few *AIRE*-expressing cells (for example, elements of the monocyte/DC lineage) ordinarily inhabiting the organs, or contaminating the preparations. However, a barely detectable *AIRE* mRNA does not imply appreciable levels of translation.

In searching for AIRE protein, greater accuracy comes from the use of monoclonal anti-AIRE Abs, especially if joined to methods, such as flow cytometry, able to improve the purity of the cell samples.

In any case, thymic localization of AIRE remains the most relevant to its function: a confirmation of the hypothesis that, by modulating thymic *AIRE* expression, we would be able to condition the susceptibility to autoimmune diseases, could delineate promising opportunities in the fight against autoimmunity. There is growing evidence that, as suggested by animal models, secondary lymphoid organs (lymph nodes and spleen) repropose in the periphery the mechanisms of central tolerance. This phenomenon needs better characterization, starting from the most accurate definition of *AIRE* and TsAg-encoding gene expression in the stromal cell lineages of the involved organs.

## Figures and Tables

**Table 1:** *AIRE* expression (*AIRE* mRNA and AIRE protein) in human extra-thymic systems, organs, and tissues.

Systems, organs, tissues	*AIRE* expression negative or negligibly positive		*AIRE* expression moderately or strongly positive	
	Techniques	Cell types	Techniques	Cell types
Bone marrow	NB [4] RT-PCR [18] IHC [18]		NB [5]	

Lymph nodes	IHC [18] FC [20]	CD14^−^ cells [20] B lymphocytes [18] T lymphocytes [18] Monocytes [18] Macrophages [18] DCs (plasmacytoid) [18] Epithelial cells [18] Endothelial cells [18]	NB [4, 5] isH [10] RT-PCR [16, 18] IHC [10, 11, 18] IF [10] FC [20]	Neutrophilic granulocytes [11] Lymphocytes [11] CD14^+^ cells [20] DCs (myeloid-lineage) [10, 18]

Spleen	NB [4] RT-PCR [16, 18] IHC [18]		NB [5] isH [10] IHC [10, 11]	Neutrophilic granulocytes [11] Lymphocytes (red-pulp) [11]

GALT			RT-PCR [18] IHC [18]	

Fetal liver			NB [4, 5] isH [10] IHC [10]	

Adult liver	NB [4] isH [10] RT-PCR [16, 18] WB [12] IHC [10, 18]			

Peripheral blood leukocytes	NB [4] RT-PCR [13, 16, 19] ICC [10, 13] FC [20]	Neutrophilic granulocytes [13] PBMCs [16] CD14^−^ cells [16] B lymphocytes [19] T lymphocytes [20] CD4^+^ T lymphocytes [13, 20] CD8^+^ T lymphocytes [19, 20] Monocytes [16, 19] DCs (myeloid-lineage) [16]	NB [5] RT-PCR [11, 13–15, 19, 20] ICC [11, 13] IF [11, 12] FC [20]	Neutrophilic granulocytes [11] PBMCs [11, 13, 19] PBLs [11, 12] B lymphocytes [20] T lymphocytes [19, 20] CD4^+^ T lymphocytes [19] CD14^+^ cells [20] Monocytes [11, 13, 14] DCs (myeloid-lineage) [13–15, 19] DCs (plasmacytoid) [15]

Skeletal muscle	RT-PCR [16, 18] IHC [18]			

Cartilage and bone	RT-PCR [16, 18] IHC [18]		RT-PCR [21]	Chondrocytes [21]

Heart and blood vessels	isH [10] RT-PCR [18] IHC [10, 18]			

Respiratory system (upper and lower tract)	isH [10] RT-PCR [16, 18] IHC [10, 18]			

Gastrointestinal system (upper tract, small and large bowel, salivary glands)	RT-PCR [16, 18] IHC [18]		NB [4]	

Endocrine glands (parathyroid glands, thyroid, pancreas, adrenal gland)	NB [4] isH [10] RT-PCR [16, 18] WB [12] IHC [10, 18]		NB [5]	

Genito-urinary system, placenta, mammary gland	isH [10] RT-PCR [16, 18] IHC [10, 18]		NB [5]	

Skin and annexes	isH [10] RT-PCR [16, 18] IHC [10, 18]		RT-PCR [22] IF [22, 23]	Keratinocytes (epidermal and HF) [23]

Central and peripheral nervous system	RT-PCR [16, 18] IHC [18]			

Eye and annexes	RT-PCR [18] IHC [18]			

*AIRE*: autoimmune regulator, GALT: gut-associated lymphoid tissue, PBMCs: peripheral blood mononuclear cells, PBLs: peripheral blood lymphocytes, DCs: dendritic cells, HF: hair-follicle, CD: cluster of differentiation, NB: Northern blotting, isH: in situ hybridization, RT-PCR: reverse transcriptase-polymerase chain reaction, WB: Western blotting, IHC: immunohistochemistry, ICC: immunocytochemistry, IF: immunofluorescence, and FC: flow cytometry.

**Table 2:** *Aire* expression (*Aire* mRNA and Aire protein) in murine extra-thymic systems, organs, and tissues.

	*Aire* expression negative or negligibly positive		*Aire* expression moderately or strongly positive	
Systems, organs, tissues	Techniques	Cell types	Techniques	Cell types
Bone marrow			isH [25] IHC [25]	Myeloblasts [25] Lymphoblasts [25] Megacaryocytes [25]

Lymph nodes	WB [26] IHC [25, 26]	Lymphocytes (germinal-center) [25]	isH [25] RT-PCR [25–27] IHC [25, 28]	Lymphocytes (germinal-center) [28] Lymphocytes (paracortical) [28] Lymphocytes (medullary) [25] DCs [25]

Spleen	NB [29, 30] isH [28] RT-PCR [30] WB [26] IHC [25, 26, 28]	Lymphocytes (red-pulp) [28] Lymphocytes (white-pulp) [25]	isH [25] RT-PCR [25–29, 31] IHC [25, 28] IF [28]	Neutrophilic granulocytes [25] Lymphocytes (red-pulp) [25] Lymphocytes (white-pulp) [28] B lymphocytes [28] T lymphocytes [28] DCs [25] DCs (myeloid-lineage) [26, 31] DCs (lymphoid-lineage) [26] Macrophages [25] Smooth-muscle cells [25]

Fetal liver			RT-PCR [25]	

Adult liver	NB [29, 30] isH [28] RT-PCR [27, 30] WB [26, 28] IHC [26, 28]		isH [25] RT-PCR [25, 26, 28] IHC [25, 32]	Hepatocytes [25, 32] Küpffer cells [25]

Peripheral blood leukocytes	RT-PCR [26]		ICC [25]	Neutrophilic granulocytes [25] PBLs [25] Monocytes [25]

Skeletal muscle	NB [29, 30] WB [26] IHC [26]		RT-PCR [26, 29]	

Heart	NB [29] isH [28] RT-PCR [27, 30] IHC [28]		RT-PCR [28, 29]	

Respiratory system (upper and lower tract)	NB [29] isH [28] RT-PCR [27] WB [26] IHC [26, 28]	Small-airway epithelial cells [28] Alveolar cells [28]	RT-PCR [28–30] IHC [25, 28]	Airway epithelial cells [25] Large-airway epithelial cells [28] Type-1 and type-2 pneumocytes [25] Alveolar macrophages [25]

Salivary glands	RT-PCR [27]		IHC [25]	Tubulo-acinar and duct cells [25]

Gastrointestinal system (upper tract)	NB [30] isH [28] RT-PCR [27]		RT-PCR [28] IHC [25]	Mucosal and glandular epithelial cells [25]

Gastrointestinal system (small intestine and large bowel)	NB [30] isH [28] IHC [28]	Enterocytes (small intestine) [28] Neuroendocrine cells [28]	RT-PCR [28] IHC [25, 28]	Mucosal and glandular epithelial cells [25] Goblet cells [28]

Hypophysis			IHC [25]	Anterior- and intermediate-lobe cells [25]

Thyroid	RT-PCR [27, 30]		IHC [25]	Follicular and parafollicular cells [25]

Pancreas	RT-PCR [26, 27, 30]		IHC [25]	Langerhans-islet cells [25] Acinar cells [25]

Adrenal gland	isH [28] RT-PCR [27, 28] WB [26, 28] IHC [26, 28]		RT-PCR [26, 30] IHC [25]	Cortical-layer cells [25] Medullary chromaffin cells [25]

Urinary system	NB [29] isH [28] RT-PCR [27, 29] WB [26] IHC [26]		isH [25] RT-PCR [25, 28, 30] IHC [25, 28]	Glomerular and tubular epithelial cells [25, 28] Urinary-tract epithelial cells [25] Bladder smooth-muscle cells [25]

Male genital system	NB [29] isH [28] WB [26] IHC [26, 28]	Mature germinal cells [28]	isH [25] RT-PCR [25, 26, 28–30] IHC [25, 28]	Germinal cells [25] Immature germinal cells [28] Sertoli cells [25] Leydig cells [25] Spermatic-tract epithelial cells [25]

Female genital system	isH [28] WB [26] IHC [26]		isH [25] RT-PCR [27, 28, 30] IHC [25, 28]	Oocytes [25] Follicular cells [25, 28] Luteal cells [25] Interstitial cells [25] Fallopian-tube epithelial cells [28] Endometrial cells [25] Myometrial cells [25]

Central and peripheral nervous system	NB [29] isH [28] RT-PCR [29]		isH [25] RT-PCR [25, 28] IHC [25, 28, 32]	Neurons of cerebral cortex, basal nuclei, brainstem nuclei, spinal cord [25, 28] Granular neurons, Purkinje cells [25, 32] Glial cells [25]

Eye and annexes	RT-PCR [27]		IHC [25]	Retinal-layer elements [25]

*Aire*: autoimmune regulator, PBLs: peripheral blood lymphocytes, DCs: dendritic cells, NB: Northern blotting, isH: in situ hybridization, RT-PCR: reverse transcriptase-polymerase chain reaction, WB: Western blotting, IHC: immunohistochemistry, ICC: immunocytochemistry, and IF: immunofluorescence.
